# A Machine Learning-Based Aging Measure Among Middle-Aged and Older Chinese Adults: The China Health and Retirement Longitudinal Study

**DOI:** 10.3389/fmed.2021.698851

**Published:** 2021-12-01

**Authors:** Xingqi Cao, Guanglai Yang, Xurui Jin, Liu He, Xueqin Li, Zhoutao Zheng, Zuyun Liu, Chenkai Wu

**Affiliations:** ^1^Department of Big Data in Health Science, School of Public Health and Center for Clinical Big Data and Analytics, Second Affiliated Hospital, Zhejiang University School of Medicine, Hangzhou, China; ^2^Global Health Research Center, Duke Kunshan University, Kunshan, China; ^3^MindRank AI ltd., Hangzhou, China

**Keywords:** biological age, disability, machine learning, mortality, aging measure

## Abstract

**Objective:** Biological age (BA) has been accepted as a more accurate proxy of aging than chronological age (CA). This study aimed to use machine learning (ML) algorithms to estimate BA in the Chinese population.

**Materials and methods:** We used data from 9,771 middle-aged and older Chinese adults (≥45 years) in the 2011/2012 wave of the China Health and Retirement Longitudinal Study and followed until 2018. We used several ML algorithms (e.g., Gradient Boosting Regressor, Random Forest, CatBoost Regressor, and Support Vector Machine) to develop new measures of biological aging (ML-BAs) based on physiological biomarkers. R-squared value and mean absolute error (MAE) were used to determine the optimal performance of these ML-BAs. We used logistic regression models to examine the associations of the best ML-BA and a conventional aging measure—Klemera and Doubal method-BA (KDM-BA) we previously developed—with physical disability and mortality, respectively.

**Results:** The Gradient Boosting Regression model performed the best, resulting in an ML-BA with an R-squared value of 0.270 and an MAE of 6.519. This ML-BA was significantly associated with disability in basic activities of daily living, instrumental activities of daily living, lower extremity mobility, and upper extremity mobility, and mortality, with odds ratios ranging from 1 to 7% (per 1-year increment in ML-BA, all *P* < 0.001), independent of CA. These associations were generally comparable to that of KDM-BA.

**Conclusion:** This study provides a valid ML-based measure of biological aging for middle-aged and older Chinese adults. These findings support the application of ML in geroscience research and may help facilitate preventive and geroprotector intervention studies.

## Introduction

Aging is a complex, inevitable, and multifactorial process, characterized by functional deterioration, physiological damage, and multiple age-related diseases (1). One key question to address aging-related issues is how to precisely quantify aging, with accumulating evidence supporting the utility of biological age (BA) in predicting age-related outcomes and differentiating individual health status (2–5). To be more specific, one study on 2,844 Chinese Singaporeans developed BA with the Klemera and Doubal method (KDM) and found that BA is better than chronological age (CA) for predicting life span (mortality) and healthspan (frailty) (2). BA has therefore been accepted as a more accurate proxy of aging than CA.

Biological age (BA) is generally referred to as a single latent variable that integrated multiple biomarkers relevant to health (6). Various statistical methods have been used to approximate BA, such as the multiple linear regression method (7), the principal component analysis (8), Hochschild's method (9), and KDM (10). KDM has been suggested as the optimal method for BA estimation (11). Although traditional methods have been demonstrated to perform well in predicting adverse aging outcomes (7–10), they may encounter obstacles when dealing with complex, multidimensional data. Among such multidimensional data, there are complex interactions among the features such as the interaction between vitamin D and albumin on mortality (12), and most of the current models were developed based on regression methods. The researcher needs to manually input the predefined interactions. Missing those complex interactions in the regression model may result in an inaccurate prediction of outcomes. Machine learning (ML) offers tremendous opportunities for researchers to address these issues (1, 13). In the prediction model developed by machine learning methods, the model can automatically identify those interactive relationship from the data and if it is unnecessary to specify interactions. Accordingly, ML methods help to figure out complicated relations among the biomarkers and generate more accurate prediction models. Also, the ML methods can reduce the loss of biological information such as the complex interactions (1). However, the application of ML in the development of aging measures has not been studied thoroughly (2, 14–17). Most of these studies were conducted among adults in Europe and the US (14–16) and ML seems to not provide more accurate aging measures than conventional methods in one study with eight biomarker features in men and 10 features in women (2). The small number of features may be one potential limitation for such methods. More studies are required to validate the application values of ML in other populations and with more features.

China is facing rapid population aging, which brings formidable challenges to policymakers and caregivers. In 2019, the Chinese population accounted for 18% of the world population, with 164.5 million adults aged 65 and over and 26 million adults aged 80 and over (18). Developing aging measures for the Chinese population is of great significance to solve aging-related issues in this large country, such as facilitating the early identification of adults at risk. To date, a few relevant studies have been conducted in the Chinese population (5, 8, 19–23). Most of them used the multiple linear regression method (19, 23) or the principal component analysis method (8, 20, 22). We have previously provided a step forward, i.e., developing a valid physiological biomarker-based aging measure using KDM (hereafter referred to as KDM-BA) (5). As the KDM measurement included limited biomarkers, we considered building the ML-based aging measurement with more features among the Chinese population and evaluating how it behaves relative to the most recent KDM-BA we developed.

Therefore, this study aimed to apply several ML algorithms (e.g., Gradient Boosting Regressor, Random Forest, CatBoost Regressor, and Support Vector Machine) to develop new aging measures (hereafter referred to as ML-BAs). We then examined the associations of the best ML-BA and KDM-BA with physical disability and mortality during the follow-up period. We used data from the China Health and Retirement Longitudinal Study (CHARLS), a nationally representative survey.

## Materials and Methods

### Study Population

**Figure 1:**
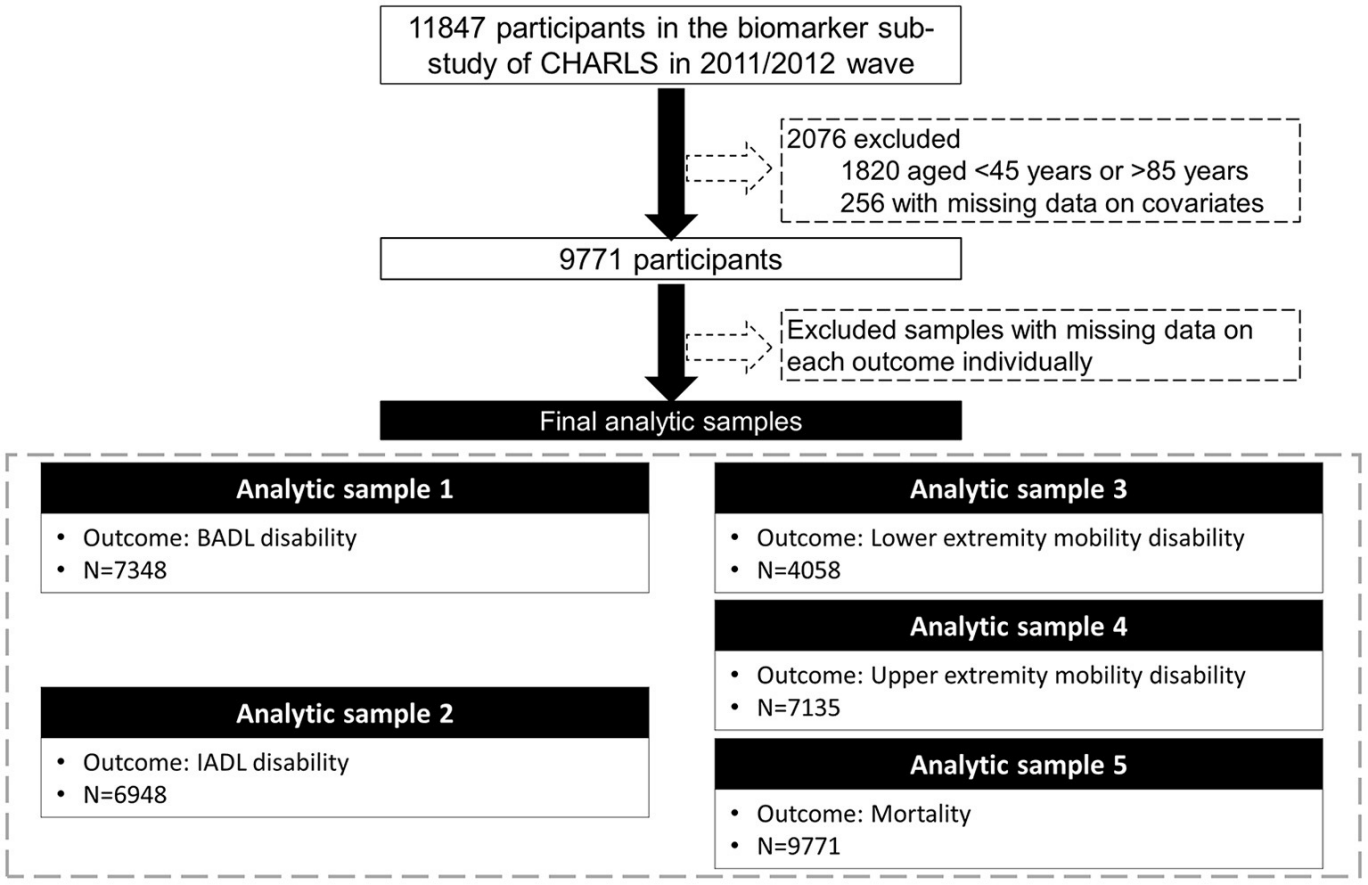
Flow chart of the analytic sample. CHARLS, the China Health and Retirement Longitudinal Study; BADL, basic activities of daily living; IADL, instrumental activities of daily living.

Data were from CHARLS, a nationally representative longitudinal survey of middle-aged and older adults (≥45 years) in China. The details of the study design and comprehensive assessments have been described elsewhere (24). In brief, CHARLS used a multistage sampling strategy covering 28 provinces, 150 counties/districts, and 450 villages/urban communities across the country. Adults aged 45 years and older were first recruited in 2011/2012, and completed three follow-up visits biennially up to 2017/2018. Ethical approval for collecting data on human subjects was received from the institutional review board at Peking University. Written informed consent was obtained from all the participants. The oldest-old population (over 85 years) was highly vulnerable to non-communicable diseases and socially disadvantaged (25). In our study, there were only 54 oldest-old participants among those who provided blood samples. Due to the small number of the oldest-old and the differences that existed between the oldest-old and the younger-old, we excluded those aged older than 85 years. Out of the 11,847 participants enrolled in the baseline survey (2011/2012) and provided blood samples, we excluded those aged <45 years or older than 85 years (*N* = 1,820), with missing data on covariates (*N* = 256), leaving the analytic sample of 9,771 adults aged 45–85 years. We then assembled various analytic samples for different outcomes due to missingness on each outcome (Figure 1).

### Biomarker Selection and BA Calculation

#### ML-BA Calculation

Candidate biomarkers were considered based on knowledge about their role in the aging process, application in previous aging studies, and data availability. A total of 16 blood biomarkers (i.e., total cholesterol, triglyceride, glycated hemoglobin, urea, creatinine, high-sensitivity C-reactive protein, platelet count, white blood cell count, mean corpuscular volume, glucose, high-density lipoprotein, low-density lipoprotein, hemoglobin, cystatin, uric acid, and hematocrit) were measured in the 2011/2012 wave of CHARLS (24), plus systolic and diastolic blood pressure, and pulse, resulting in 19 candidate biomarkers for the initial consideration in this study. We first imputed the missing data with the mean and normalized data using a min-max scalar, because data imputation and normalization were the necessary steps in the process of ML (26, 27). Imputing missing values contributed to the improved predictive power regardless of the conditions of missingness (26). Training models with normalized data usually helped to enhance performance; thus data normalization was an essential step in ML as well (27). Then, we trained models with these 19 candidate biomarkers using 10-fold cross-validations to obtain the R-squared value and the mean absolute error (MAE). We trained these models using default parameters that have been pre-defined by python package providers, to avoid randomness in the process of personnel adjustment. Almost all classic ML methods that can perform regression analysis were considered in our work. The top seven performers included Gradient Boosting Regressor, Light Gradient Boosting Machine, CatBoost Regressor, Random Forest, Extra Trees Regressor, Support Vector Machine, and AdaBoost Regressor. The final ML-BA in the unit of years was computed.

#### KDM-BA Calculation

Following the procedures we previously described (5, 28, 29), the 19 candidate biomarkers above were considered. Some sets of biomarkers were highly correlated, such as systolic and diastolic blood pressure. According to Klemera and Doubal (10), and considering the use of biomarkers in clinical settings and their property, we kept one for each set in the biomarkers list. We then selected eight biomarkers that showed an absolute age correlation >0.1. The final list included total cholesterol, triglyceride, glycated hemoglobin, urea, creatinine, high-sensitivity C-reactive protein, platelet count, and systolic blood pressure, representing various domains of the physical function: cardiac-metabolic function (total cholesterol, triglyceride, glycated hemoglobin, and systolic blood pressure), kidney function (urea, creatinine), and immune function (high-sensitivity C-reactive protein, and platelet count). The log transformations of non-normally distributed biomarkers (e.g., high-sensitivity C-reactive protein) were performed prior to the calculation of KDM-BA. Then, the KDM takes information from the *m* number of regression lines of the CA regressed on the *m* biomarkers (*m* = eight in this study) briefly. The final product is the KDM-BA in the unit of years.

### Physical Disability

The physical function status of the basic activities of daily living (BADL) was assessed based on six daily activities, including eating, dressing, transferring, using the toilet, bathing, and continence (30). The participants were asked if they needed assistance with each of the activities. We categorized the participants as having BADL disability if they had incontinence problems or needed assistance in performing at least one of the other five activities (eating, dressing, transferring, toileting, and bathing) (31). The physical function status of the instrumental activities of daily living (IADL) was assessed by five instrumental activities, including cleaning the house, managing money, taking medications, shopping for groceries, and preparing a hot meal (32). We categorized participants as having IADL disability if they needed assistance in performing at least one of the five instrumental activities (31). Mobility function was divided into the function of upper extremities and lower extremities. The mobility function of the upper extremity was assessed by three activities, including extending arms up, lifting 10 jin (i.e., 5 kg), and picking up a small coin. The mobility function of the lower extremity was assessed by four activities, including walking 100 m, climbing several flights of stairs, getting up from a chair, and stooping or kneeling or crouching. We categorized participants as having mobility disability if they needed assistance in performing at least one activity (33). The functional status was assessed at baseline, 2013 wave, and 2015 wave. Since the time of developing disability during the follow-up period was not available, we constructed a binary outcome to denote the occurrence of disability within the 4-year follow-up since baseline.

### Mortality

In CHARLS, the death information was collected from the exit interviews in the 2013, 2015, and 2018 waves. However, in the 2015 and 2018 waves, the exact date of death was not available. Therefore, in this study, we constructed a binary variable to denote the occurrence of death within the 6-year follow-up since baseline, as we did before (33).

### Covariates

All covariates were obtained at baseline. The sociodemographic variables including age, sex, educational level, marital status, and residence were collected from the self-reported questionnaire. The educational level was defined as no school, primary school, middle school, and high school or above. The marital status was defined as currently married and others (e.g., separated, divorced, widowed). The residence was defined as urban and rural. Health behaviors including smoking, alcohol drinking, and body mass index (BMI) (kg/m^2^) were collected through the structured home interview. Smoking status was defined as current smoker and non-smoker. Alcohol drinking status was defined as current drinker and non-drinker. The BMI was calculated as weight in kilograms divided by height in meters squared. We categorized participants as underweight (BMI < 18.5 kg/m^2^), normal (18.5 ≤ BMI < 24.0 kg/m^2^), overweight (24.0 ≤ BMI < 28.0 kg/m^2^), and obese (BMI ≥ 28 kg/m^2^). The disease count was determined by counting 10 self-reported chronic diseases, including hypertension, diabetes or high blood sugar, cancer or malignant tumor, chronic lung disease, heart problems, stroke, kidney disease, stomach or other digestive diseases, arthritis or rheumatism, and asthma. We then divided participants into four groups—no disease, one disease, two diseases, and three or more diseases.

### Statistical Analyses

We used 10-fold cross-validations to train ML-BA with a 90% training dataset and validate it with a 10% testing dataset. We compared different ML algorithms based on the R-squared value and the MAE. Finally, we selected the Gradient Boosting Regression model to compute the best ML-BA in the unit of years in the total population. To estimate the relative importance of biomarkers for the two aging measures, we calculated the SHapley Additive exPlanations (SHAP) value and the R-squared value of the biomarkers for ML-BA and KDM-BA, respectively.

The baseline characteristics of the study population were presented as means ± SD for the continuous variables or numbers (percentages) for the categorical variables. To examine the associations of the two aging measures (i.e., ML-BA and KDM-BA) with 4-year physical disability incidence and 6-year mortality risk, we used logistic regression models. Odds ratios (ORs) and corresponding 95% confidence intervals (CIs) were documented. Two models were used in our study. Model 1 was a crude model, whereas model 2 was adjusted for CA.

All statistical analyses were performed using SAS version 9.4 (SAS Institute, Cary, NC, USA), Stata version 15 (Stata Corp, College Station, Texas, USA), and Python version 3.8.3. *P* < 0.05 (two-tailed) was considered statistically significant.

## Results

The basic characteristics of the study population are presented in Table 1. The mean CA of the study population was 59.1 (SD = 9.2) years. Of the 9,771 middle-aged and older adults, ~44.6% were aged ≥ 60 years, 53.5% were women. The mean CAs of men and women was 59.8 (SD = 9.1) years and 58.5 (SD = 9.2) years, respectively.

**Table 1:** Baseline characteristics of the study population.

	**Total**	**Male**	**Female**
	**(*N* = 9,771)**	**(*N* = 4,545)**	**(*N* = 5,226)**
Age, years	59.1 ± 9.2	59.8 ± 9.1	58.5 ± 9.2
<60 years	5,414 (55.4)	2,361 (52.0)	3,053 (58.4)
≥60 years	4,357 (44.6)	2,184 (48.1)	2,173 (41.6)
ML-BA	59.4 (5.8)	60.0 (5.8)	58.8 (5.8)
KDM-BA	57.0 (9.9)	58.2 (9.4)	56.1 (10.3)
Sex, female	5,226 (53.5)	–	–
Residence, rural	6,366 (65.2)	3,005 (66.1)	3,361 (64.3)
Education				
No schooling	2,882 (29.5)	601 (13.2)	2,281 (43.7)
Primary school	4,018 (41.1)	2,182 (48.0)	1,836 (35.1)
Middle school	1,923 (19.7)	1,160 (25.5)	763 (14.6)
High school or more	948 (9.7)	602 (13.3)	346 (6.6)
Marital status				
Currently married	8,156 (83.5)	3,984 (87.7)	4,172 (79.8)
Others	1,615 (16.5)	561 (12.3)	1,054 (20.2)
Smoking status				
Non-smoker	6,797 (69.6)	1,897 (41.7)	4,900 (93.8)
Smoker	2,974 (30.4)	2,648 (58.3)	326 (6.24)
Alcohol consumption				
Non-drinker	5,973 (61.1)	1,522 (33.5)	4,451 (85.2)
Drinker	3,798 (38.9)	3,023 (66.5)	775 (14.8)
BMI (kg/m^2^)	23.5 ± 3.9	23.0 ± 3.6	24.0 ± 4.1
BMI category^*^				
Underweight	650 (6.8)	315 (7.1)	335 (6.5)
Normal	4,990 (52.0)	2,627 (58.8)	2,363 (46.1)
Overweight	2,828 (29.5)	1,149 (25.7)	1,679 (32.7)
Obese	1,130 (11.8)	377 (8.4)	753 (14.7)
Disease counts				
0	2,938 (30.1)	1,469 (32.3)	1,469 (28.1)
1	3,110 (31.8)	1,482 (32.6)	1,628 (31.2)
2	2,132 (21.8)	943 (20.8)	1,189 (22.8)
3	1,591 (16.3)	651 (14.3)	940 (18.0)

*ML-BA, Machine Learning method-biological age; KDM-BA, Klemera and Doubal method-biological age; BMI, body mass index. The continuous variables and categorical variables were expressed as mean ± standard deviation, and number (percentage), respectively.*

* *BMI was calculated as weight in kilograms divided by height in meters squared. Underweight was defined as BMI < 18.5 kg/m^2^; normal was defined as 18.5 ≤ BMI < 24.0 kg/m^2^; overweight was defined as 24.0 ≤ BMI < 28.0 kg/m^2^; and obese was defined as BMI ≥ 28 kg/m^2^*.

### Characteristics of ML-BA

We considered Gradient Boosting Regressor, Light Gradient Boosting Machine, CatBoost Regressor, Random Forest, Extra Trees Regressor, Support Vector Machine, and AdaBoost Regressor in our study. The R-squared value of models ranged from 0.217 to 0.270, and the MAE of the models ranged from 6.619 to 6.877 (Table 2). Among them, the Gradient Boosting Regressor model performed best with the highest R-squared value of 0.270 and the lowest MAE of 6.519. Hence, we computed ML-BA using the Gradient Boosting Regression model with 19 biomarkers.

**Table 2:** MAE, MSE, RMSE, and R-squared value of machine learning models.

**Model**	**MAE**	**MSE**	**RMSE**	***R*** **-squared**
				**value**
Gradient boosting regressor	6.519	64.127	8.001	0.270
Light gradient boosting machine	6.532	64.875	8.049	0.261
CatBoost regressor	6.527	65.121	8.063	0.258
Random forest	6.557	65.126	8.065	0.258
Extra trees regressor	6.576	65.330	8.075	0.256
Support vector machine	6.655	68.141	8.248	0.224
AdaBoost regressor	6.877	68.804	8.289	0.217

*MAE, Mean Absolute Error; MSE, Mean Square Error; RMSE, Root Mean Square Error*.

**Figure 2:**
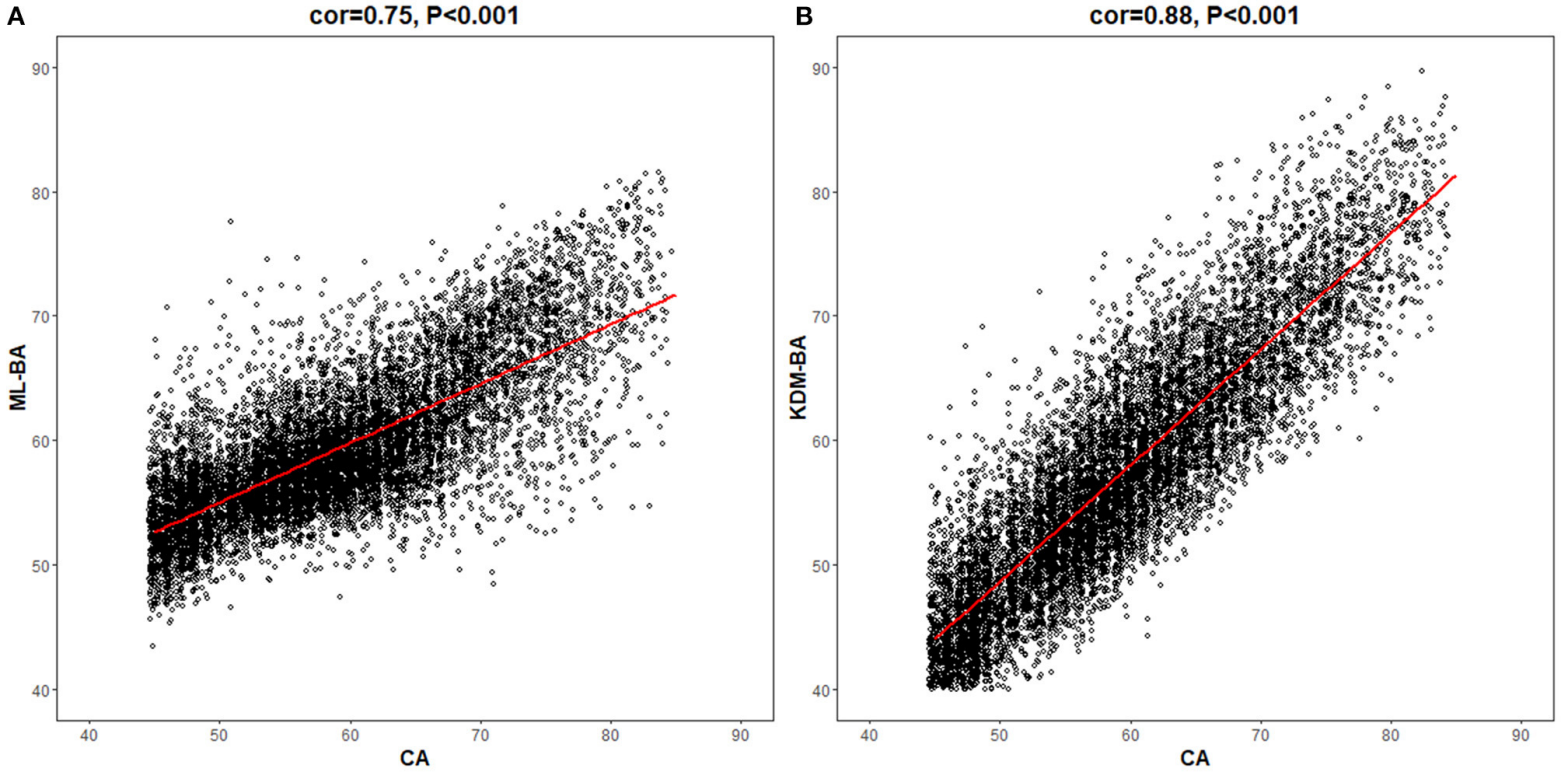
Correlations of chronological age with machine learning method-biological age and Klemera and Doubal method-biological age. CA, chronological age; ML-BA, Machine Learning method-biological age; KDM-BA, Klemera and Doubal method-biological age. **(A)** and **(B)** show the correlation between CA and the two measures (ML-BA and KDM-BA), respectively.

In the total study population, the ML-BA ranged from 43 to 82 years, with a mean of 59.4 (SD = 5.8) years. In men, the ML-BA ranged from 47 to 82 years, with a mean of 60.0 (SD = 5.8) years. In women, the ML-BA ranged from 43 to 81 years, with a mean of 58.8 (SD = 5.8) years. As shown in Figure 2, ML-BA and CA were highly correlated (cor = 0.75).

### The Importance of Biomarkers for ML-BA and KDM-BA

As suggested in Supplementary Figure 1, cystatin, systolic blood pressure, diastolic blood pressure, mean corpuscular volume, hemoglobin, and urea were the top six important biomarkers for ML-BA. Interestingly, systolic blood pressure and urea were also the top important biomarkers for KDM-BA (Supplementary Table 1). Similarly, triglyceride, platelet count, and creatinine were the least important biomarkers for both ML-BA and KDM-BA.

### Associations of ML-BA and KDM-BA With a Physical Disability

As shown in Table 3, both ML-BA and KDM-BA were significantly associated with 4-year physical disability in the full sample. In the crude model, each 1-year increment in ML-BA increased the odds of disability in BADL, IADL, lower extremity mobility, and upper extremity mobility by 6% (OR = 1.06, 95% CI = 1.05, 1.07), 6% (OR = 1.06, 95% CI = 1.05, 1.07), 4% (OR = 1.04, 95% CI = 1.03, 1.05), and 7% (OR = 1.07, 95% CI = 1.06, 1.08), respectively. The strength of these associations was slightly stronger compared with that of KDM-BA. For example, each 1-year increment in KDM-BA increased the odds of disability in the upper extremity mobility by 4% (OR = 1.04, 95% CI = 1.03, 1.05). Further subgroups analyses by sex did not change the results substantially.

**Table 3:** Unadjusted associations of CA, ML-BA, or KDM-BA with disability and mortality in the full sample and sex subgroup.

		**BADL disability**	**IADL disability**	**Lower extremity**	**Upper extremity**	**Mortality**
				**mobility disability**		
		**OR (95% CI)**	**OR (95% CI)**	**OR (95% CI)**	**OR (95% CI)**	**OR (95% CI)**
No. of events/No. of participants		1,860/7,797	1,935/7,490	2,380/4,375	1,947/7,698	882/9,771
Total	CA only	1.048 (1.042, 1.054)	1.045 (1.039, 1.051)	1.03 (1.02, 1.04)	1.05 (1.04, 1.06)	1.11 (1.10, 1.12)
	ML-BA only	1.06 (1.05, 1.07)	1.06 (1.05, 1.07)	1.04 (1.03, 1.05)	1.07 (1.06, 1.08)	1.16 (1.14, 1.17)
	KDM_BA only	1.043 (1.037, 1.048)	1.037 (1.031, 1.043)	1.024 (1.017, 1.031)	1.04 (1.03, 1.05)	1.104 (1.096, 1.113)
Male	CA only	1.06 (1.05, 1.07)	1.06 (1.05, 1.07)	1.04 (1.03, 1.05)	1.05 (1.04, 1.04)	1.10 (1.09, 1.12)
	ML-BA only	1.07 (1.06,1.09)	1.08 (1.07,1.10)	1.06 (1.05, 1.07)	1.08 (1.06,1.09)	1.14 (1.13, 1.16)
	KDM-BA only	1.06 (1.05, 1.07)	1.05 (1.04, 1.06)	1.04 (1.03,1.05)	1.05 (1.04, 1.06)	1.10 (1.09, 1.11)
Female	CA only	1.046 (1.038, 1.054)	1.043 (1.035, 1.051)	1.03 (1.02,1.04)	1.055 (1.047, 1.064)	1.13 (1.11, 1.14)
	ML-BA only	1.06 (1.05, 1.08)	1.06 (1.05, 1.07)	1.04 (1.02,1.05)	1.08 (1.06, 1.09)	1.17 (1.15, 1.19)
	KDM-BA only	1.04 (1.03, 1.05)	1.036 (1.029, 1.044)	1.03 (1.02, 1.04)	1.045 (1.037, 1.052)	1.11 (1.10, 1.12)

*CA, chronological age; ML-BA, Machine Learning method-biological age; KDM-BA, Klemera and Doubal method-biological age; BADL, basic activities of daily living; IADL, instrumental activities of daily living; OR, odds ratio; CI: confidence interval.*

*Participants with prevalent disability in BADL/IADL/lower extremity mobility/upper extremity mobility were excluded for analyses of BADL/IADL/lower extremity mobility/upper extremity mobility, respectively*.

Table 4 shows the associations of ML-BA and KDM-BA with a physical disability when adjusting for CA in the full sample. ML-BA was still significantly associated with all functional disabilities, with ORs ranging from 1.01 to 1.02. Significant association of KDM-BA with disability in BADL was observed, with OR of 1.01 (95 % CI = 1.00, 1.03).

**Table 4:** Risk estimates of physical disability and mortality predicted by ML-BA and KDM-BA adjusting for CA.

**Model**	**Variable**	**BADL disability**	**IADL disability**	**Lower extremity**	**Upper extremity**	**Mortality**
				**mobility disability**	**mobility disability**	
		**OR (95% CI)**	**OR (95% CI)**	**OR (95% CI)**	**OR (95% CI)**	**OR (95% CI)**
No. of events/No. of participants		1,860/7,797	1,935/7,490	2,380/4,375	1,947/7,698	882/9,771
CA+ ML-BA	CA	1.04 (1.03, 1.05)	1.04 (1.03, 1.05)	1.02 (1.01, 1.03)	1.04 (1.03, 1.05)	1.08 (1.06, 1.09)
	ML-BA	1.01 (1.00, 1.03)	1.02 (1.00, 1.03)	1.02 (1.00, 1.03)	1.02 (1.01, 1.03)	1.07 (1.05, 1.09)
CA+ KDM-BA	CA	1.04 (1.02, 1.05)	1.04 (1.03, 1.06)	1.03 (1.01, 1.04)	1.05 (1.03, 1.06)	1.06 (1.05, 1.08)
	KDM-BA	1.01 (1.00, 1.03)	1.00 (0.99, 1.01)	1.00 (0.99, 1.02)	1.00 (0.99, 1.01)	1.05 (1.04, 1.07)

*CA, chronological age; ML-BA, machine learning method-biological age; KDM-BA, Klemera and Doubal method-biological age; BADL, basic activities of daily living; IADL, instrumental activities of daily living; OR, odds ratio; CI: confidence interval.*

*articipants with prevalent disability in BADL/IADL/lower extremity mobility/upper extremity mobility were excluded for analyses of BADL/IADL/lower extremity mobility/upper extremity mobility, respectively*.

### Associations of ML-BA and KDM-BA With Mortality

Table 3 presents the associations of ML-BA and KDM-BA with 6-year mortality in full sample and subgroups by sex. Both ML-BA and KDM-BA were positively associated with mortality risk. The results of the association between KDM-BA and mortality were previously reported (5). In the full sample, each 1-year increment in ML-BA and KDM-BA increased the risk of mortality risk by 16% (OR = 1.16, 95% CI = 1.14, 1.17) and 10% (OR = 1.104, 95% CI = 1.096, 1.113), respectively. When stratified by sex, the ORs of ML-BA for mortality risk ranged from 1.14 to 1.17, consistent with that in the full sample (OR = 1.16). Similar results were found for KDM-BA.

After adjusting for CA, both ML-BA and KDM-BA were significantly associated with 6-year mortality risk, although the strength of the associations was attenuated. Each 1-year increment in ML-BA and KDM-BA increased the risk of mortality by 7% (OR = 1.07, 95% CI = 1.05, 1.09) and 5% (OR = 1.05, 95% CI = 1.04, 1.07), respectively (Table 4). The results suggested that they capture something above and beyond what can be explained by CA alone when predicting mortality.

## Discussion

In this study, we successfully developed an aging measure using the Gradient Boosting Regression model in a sample of middle-aged and older Chinese adults. We found that this ML-BA was predictive of physical disability and mortality during the follow-up period, and these associations were independent of CA. The results were better than that of KDM-BA, supporting the development of ML-BA. This ML-BA may serve as a proxy of life span in geroscience research and help with the risk stratification in the general Chinese older adults.

To date, several studies have shown that BA calculated using ML has the predictive ability for mortality risk in populations from different countries, such as the US (15, 17), Italy (34), and Singapore (2). Because of differences in genetic and socio-environmental factors, the findings may not be generalizable to various populations in other countries, such as the Chinese population, a rapidly increasing segment worldwide. To the best of our knowledge, no studies have been performed to develop BAs using ML and evaluate the associations of ML-BAs with adverse outcomes in the Chinese population. We filled up this gap in this study. More importantly, we demonstrated that the best ML-BA performed just as well as KDM-BA, which has been regarded as the best biological aging measure (28). The findings support that ML could be used to develop measures of biological aging. Moreover, both ML-BA and KDM-BA could be developed across various populations separately, and they may capture something underlying the aging process.

It should be noted that the strength of the associations of the best ML-BA with physical disability and mortality is slightly stronger than that for KDM-BA. The ML-BA in our study was computed based on 19 biomarkers, while the KDM-BA was computed based on only eight of the 19 biomarkers. The remaining 11 biomarkers included diastolic blood pressure, pulse, white blood cell count, mean corpuscular volume, glucose, high-density lipoprotein, low-density lipoprotein, hemoglobin, cystatin, uric acid, and hematocrit, which have been demonstrated to be associated with aging (35–39). Hence, we assume that the better performance of ML-BA may be due to the more information covered by ML-BA than that by KMD-BA. The ML-BA was developed without prior assumptions and was not dependent on intermediate results from multiple linear regression models (40), allowing ML-BA to be easily verified. In general, ML-BA may therefore provide a useful tool to identify individual risks for adverse outcomes.

The stable associations of ML-BA and KDM-BA with physical disability and mortality risk can be interpreted by looking into the biological biomarkers used to develop the two aging measures. The aging process is subclinical, characterized by various types of biological degradations. So, it is proposed to estimate aging based on cellular and molecular hallmarks (41). In our study, the biomarkers used for ML-BA and KDM-BA computation represent different but important domains of physiological function or systems: immune system (e.g., high-sensitivity C-reactive protein, platelet count, and white blood cell), cardiac-metabolic system (e.g., Total cholesterol, systolic blood pressure, and low-density lipoprotein), and kidney system (e.g., urea, creatinine, cystatin, and uric acid). First, the immune system is a homeostatic system that helps to maintain the function of the organisms, and age-related changes in immune function have been demonstrated to affect longevity (42). Due to infectious diseases, older adults usually have an increased risk of morbidity and mortality (43), emphasizing the importance of maintaining the function of the immune system during the aging process. Second, since the incidence of heart disease increases sharply with age, it has been postulated that aging and cardiovascular disease are interrelated (44) and may share common pathology (45). During the normal aging process, the cardiac-metabolic function is impaired with the increase of age (44), contributing to adverse age-related outcomes. Finally, evidence has suggested that even in the absence of comorbidities, the kidney may experience significant age-related changes in structure and function (46). This implies that the deterioration of kidney function may be one of the important phenotypes of the aging process. The aging measures we developed in the current study integrated various biomarkers of immune function, cardiac-metabolic function, and kidney function; therefore, they could reflect the aging process through multiple physiological systems and work well in predicting physical disability and mortality.

From the perspective of the application, both ML-BA and KDM-BA could be considered since they had satisfactory predictive performance in this study. The choice of methods is largely dependent on sample size, distribution, and data availability. ML is more non-parametric and modeling-based, while KDM is more parametric and theory-based. Non-parametric methods do not require assumptions about sample size and data distribution (47, 48), and thus, are flexible. Therefore, ML-BA is a good choice if the shape of the distribution was not suited for parametric methods. On the contrary, KDM-BA would be efficient in a sound dataset.

In this study, the large sample size of the nationwide prospective cohort study provided us with the opportunity to develop aging measures by ML and explore its associations with adverse health outcomes in middle-aged and older Chinese adults. Nevertheless, limitations in this study should be acknowledged. First, the relatively short follow-up period (i.e., up to 6 years) of the CHARLS has impeded us to explore the long-term effect of aging measures on the outcomes. Longitudinal studies with long-term follow-up are needed to confirm the associations. Second, we did not have data on the exact timing of physical disability incidence and death. Therefore, we cannot evaluate the impact of BAs on survival time and we used the 2-year mortality as an alternative. Third, we did not have data on the incidence of chronic diseases (e.g., diabetes, heart disease, and stroke), impeding us to evaluate the associations of biological aging with chronic diseases. Fourth, it would be useful to validate the predictive performance of ML-BA in another dataset. However, there are only a few large aging cohort studies in China, such as the Chinese Longitudinal Healthy Longevity Survey (CLHLS), the China Health and Nutrition Survey (CHNS), and the CHARLS, which was used in our work. The CLHLS and CHNS do not have all the biomarkers used in our work, we were unable to validate this ML-BA in this regard. Finally, the utility of this ML-BA needs to be further validated as it needs more input information. However, with the further development of medical informatization, more individual-level data will be available and this method will be the workflow for building the BA with more information.

In summary, this study provides a valid ML-based measure of biological aging for middle-aged and older Chinese adults. We further demonstrated that this ML-BA was associated with physical disability incidence and mortality. These associations were comparable with that of KDM-BA, a valid physiological biomarker-based aging measure we have previously developed. The findings support the application of ML in geroscience research and promote further understanding of the aging process. Together with KDM-BA, these aging measures could serve as a proxy of life span and help with the risk stratification in the general Chinese older adults.

## Data Availability Statement

The datasets for this study can be found in the CHARLS website at http://charls.pku.edu.cn/index/en.html.

## Ethics Statement

The studies involving human participants were reviewed and approved by the Institutional Review Board at Peking University. The patients/participants provided their written informed consent to participate in this study.

## Author Contributions

ZL and CW conceived and designed the study and contributed to the critical revision of the manuscript for important intellectual contents. XC, GY, and XJ performed the analysis and wrote the initial draft of the manuscript. LH, XL, ZZ, ZL, and CW helped to interpret the results and edit the manuscript. All authors have read and agreed to the final version of the manuscript.

## Funding

The research results of this article are sponsored by the Kunshan Municipal Government research funding.

## Conflict of Interest

XJ is employed by MindRank AI ltd. The remaining authors declare that the research was conducted in the absence of any commercial or financial relationships that could be construed as a potential conflict of interest.

## Publisher's Note

All claims expressed in this article are solely those of the authors and do not necessarily represent those of their affiliated organizations, or those of the publisher, the editors and the reviewers. Any product that may be evaluated in this article, or claim that may be made by its manufacturer, is not guaranteed or endorsed by the publisher.

## Abbreviations

BA: biological age
BADL: basic activities of daily living
BMI: body mass index
CA: chronological age
CHARLS: the China Health and Retirement Longitudinal Study
CHNS: the China Health and Nutrition Survey
CI: confidence interval
CLHLS: the Chinese Longitudinal Healthy Longevity Survey
IADL: instrumental activities of daily living
KDM: the Klemera and Doubal method
KDM-BA: the Klemera and Doubal method based biological age
MAE: mean absolute error
ML: machine learning
ML-BA: the machine learning based biological age
OR: odds ratio
SD: standard deviation
SHAP: SHapley Additive exPlanations.
