# Supplementary material for: A Machine Learning-Based Aging Measure Among Middle-Aged and Older Chinese Adults: The China Health and Retirement Longitudinal Study
Source: Front Med (Lausanne). 2021 Dec 1;8:698851. doi: 10.3389/fmed.2021.698851 (PMC8671693; doi:10.3389/fmed.2021.698851)
Supplement: Supplementary file 1 [file Table_1.DOCX]

**Table S1. The R-squared values of biomarkers for KDM-BA.**

| **Biomarkers** | **R-squared value** |
| --- | --- |
| SBP | 0.193 |
| Urea | 0.060 |
| hs-CRP | 0.055 |
| HbA1c | 0.052 |
| TC | 0.051 |
| Crea | 0.036 |
| PLT | 0.010 |
| TG | 0.010 |

Notes: SBP, systolic blood pressure; hs-CRP, high-sensitivity C-reactive protein; HbA1c, glycated hemoglobin; TC, total cholesterol; Crea, creatinine; PLT, platelet count; TG, triglyceride.


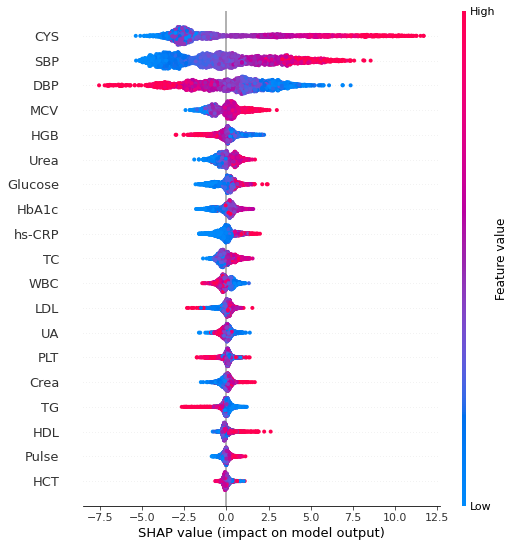


**Figure S1. The SHAP values and feature values of biomarkers for ML-BA.**

Notes: CYS, cystatin; SBP, systolic blood pressure; DBP, diastolic blood pressure; MCV, mean corpuscular volume; HGB, hemoglobin; HbA1c, glycated hemoglobin; hs-CRP, high-sensitivity C-reactive protein; TC, total cholesterol; WBC, white blood cell count; LDL, low-density lipoprotein; UA, uric acid; PLT, platelet count; Crea, creatinine; TG, triglyceride; HDL, high-density lipoprotein; HCT, hematocrit.
